# Ionic Fragmentation of the Halothane Molecule Induced by EUV and Soft X-ray Radiation

**DOI:** 10.1021/acs.jpca.4c04341

**Published:** 2024-08-23

**Authors:** A. C. F. Santos, C. A. Lucas, A. F. Lago, R. R. Oliveira, A. B. Rocha, G. G. B. de Souza

**Affiliations:** † Instituto de Física, Universidade Federal do Rio de Janeiro (UFRJ), Ilha do Fundão, Rio de Janeiro 21949-900, RJ, Brazil; ‡ Instituto de Química, Universidade Federal Fluminense, Outeiro de São João Batista s/n, Campus do Valonguinho, Niterói 24020-141, Brazil; § Centro de Ciências Naturais e Humanas, Universidade Federal do ABC (UFABC), Av. dos Estados, 5001, Santo André 09210-580, Sao Paulo, Brazil; ∥ Instituto de Química, Universidade Federal do Rio de Janeiro (UFRJ), Ilha do Fundão, Rio de Janeiro 21949-900, RJ, Brazil

## Abstract

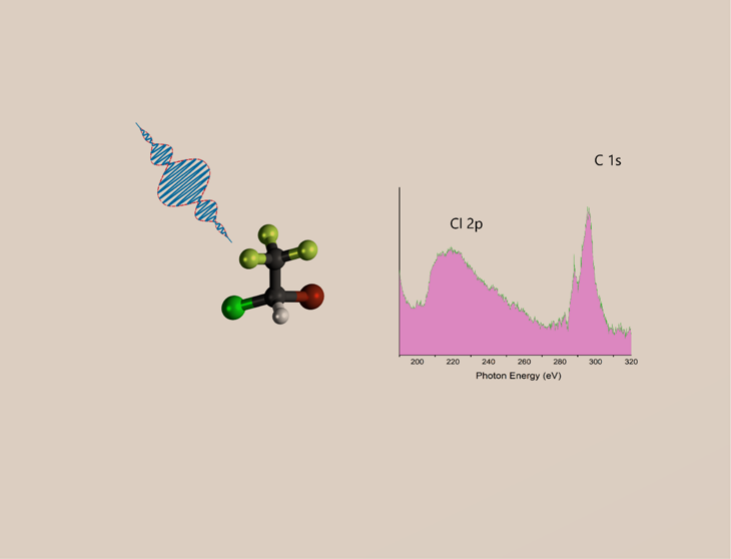

EUV and soft X-ray-induced photofragmentation of the halothane (CF_3_CHBrCl) molecule has been investigated using time-of-flight mass spectrometry in the coincidence mode (PEPICO) covering the valence region and vicinity of the bromine 3d, chlorine 2p, and carbon 1s edges. Total and partial ion yields have been recorded as a function of photon energy. At lower photon energies, the heavier singly charged molecular fragments predominate in the mass spectra. On the other hand, there is a strong tendency to the atomization of the molecule at higher photon energies. Despite the different chemical environments experienced by the two carbon atoms, weak site-specific fragmentation is observed. In addition, *ab initio* quantum mechanical calculations at the MP2 level and a series of computations with multiconfigurational self-consistent field have been performed to describe the inner-shell states.

## Introduction

A single photon, in the soft X-ray regime, is capable of exciting or ionizing an inner-shell electron in an atom or a molecule, creating an inner-shell hole. This inner-shell vacancy is normally occupied by a decaying electron from an outer shell, typically within ∼10^–15^ s time scale (depending on the energy levels involved), resulting in the ejection of either an Auger electron, which is the prevailing process in the case of low-Z atoms, or a fluorescence photon, otherwise.^1−10^ In molecules, such processes can also induce the creation of ionic, repulsive states, thus giving rise to intense molecular fragmentation.^1−10^

The localization of a core vacancy in a molecular system is known to strongly influence the fragmentation profile. The specificity in molecular fragmentation^3−10^ may be achieved by the so-called “element-specific,” “site-specific,” and “state-specific” excitation. In “element-specific fragmentation,” for example, distinct elements in the same molecule can be selectively excited such as, for instance, the Br, Cl, and C atoms in the halothane (CF_3_CHBrCl) molecule. On the other hand, “site-specific” fragmentation may be reached by the excitation of different atoms of the same element in distinct chemical environments—for instance, considering the two carbon atoms in halothane, one is bonded to H, Br, and Cl atoms [here denoted C(1)], while the other is bonded to three fluorine atoms [C(2)]. The different chemical environments experienced by these carbon atoms are expected to result in two absorption peaks at distinct energies in the C K-edge photoabsorption spectrum, corresponding to the so-called chemical shift effect.^2−7^ Therefore, knowledge of the effects of core-induced photoionization is important because it may provide tools to not only understand but also control chemical reactions. However, it should be noted that, in general, only a small fraction of the fragmentation patterns is specific to the excitation site. Finally, in the “state-specific” fragmentation,^11^ one can excite different states in the same site—for example, the Cl 2p_3/2_ and Cl 2p_1/2_ in the halothane.

There has been considerable interest in the photoexcitation, photoionization, and ion dissociation dynamics of halogenated hydrocarbons^12^ and chlorocarbons^13^ with reference to their diversity of bond-breaking routes and the investigation of possible ways of selectively controlling their fragmentation pattern.

Halothane (CF_3_CHBrCl), also known as Fluktan and Fluorotane,^14^ is a highly halogenated and volatile ethane derivative broadly used as an anesthetic agent. While one of the carbon atoms is bonded to H, Cl, and Br atoms, the other carbon atom is bonded to a CF_3_ group, resulting in two carbon atoms belonging to the same molecule, but each one is immersed in a very different chemical environment. In its electronic ground state, halothane shows *C*_1_ symmetry. The electronic configuration of the valence orbitals of the X^1^A ground state is^14^ (41a)^2^ (42a)^2^(43a)^2^(44a)^2^(45a)^2^ (46a)^2^. The lowest vertical ionization energies are^14,15^ 11.2 eV (46a)^−1^, 11.5 eV (45a)^−1^, 12.2 eV (44a)^−1^, and 12.3 eV (43a)^−1^. Halothane is a compelling system to study due to its low symmetry and its multiple edges (C, F, Br, and Cl). The worldwide expenditure for halothane is not accurately known, but there is an estimate of roughly 10^4^ kg/year.^14^ Halothane is an ozone-depleting substance with an ozone depletion potential (ODP) of 1.56, accounting for 1% of the total stratospheric ozone layer depletion.^16^ In addition, the Br atom is forecasted to be much more efficient in the depletion of ozone in the stratosphere than the Cl atom.^17^

Ferreira da Silva et al.^14^ studied the neutral electronic transitions halothane by using high-resolution VUV photoabsorption and electron energy-loss spectroscopy techniques coupled to *ab initio* theoretical calculations. Pitzer et al.^18^ studied the X-ray single-photon ionization and fragmentation of halothane by using the cold target recoil ion momentum spectroscopy technique. The authors investigated the site-selective excitation of the two carbon atoms for four-body fragmentations and, in the case of double ionization, leading to two-body fragmentation.

In the present work, new results on the photoionization and ion dissociation dynamics for the halothane molecule are presented. The reasons to select this molecule for our studies were mainly 2-fold: first, it is an interesting halocarbon polyatomic molecule containing several distinct atoms. This fact opens the possibility of probing the selective excitation/ionization processes and studying their effects on the fragmentation processes. The second reason is related to the environmental relevance of this class of molecules due to their potential role in ozone depletion schemes. We have investigated the valence, Br 3d, Cl 2p, and C 1s photoionization regions of the C_2_HClBrF_3_ molecule. The questions of interest from the point of view of the present study are (i) How does the fragmentation of the core-excited molecule change as a function of the incident photon energy (in other words, how does the fragmentation pattern change as we probe different core edges)? (ii) How efficient is the production of multiply charged ions as we move on from one core edge to another? (iii) What are the ion dissociation mechanisms? (iv) In a molecule containing three different halogen atoms, can element-specific or site-specific fragmentation mechanisms be observed? The experimental results have been obtained using the photoelectron–photoion coincidence (PEPICO) technique. *Ab initio* theoretical calculations were done to help in the assignment of the electronic transitions in several absorption edges and to characterize the bonding or antibonding character of the participant orbitals.

## Experimental Section

Fragmentation of the halothane molecule has been studied using synchrotron radiation as an ionizing agent. Analyses of the fragments were performed with the aid of time-of-flight mass spectrometry and electron–ion coincidence techniques. The experimental setup has been described elsewhere.^19^ Briefly, the experiment was performed at the Laboratório Nacional de Luz Sncrotron (LNLS-CNPEM), Campinas, São Paulo, Brazil. Light from a toroidal grating monochromator (TGM) beamline (12–310 eV) crosses a molecular gas jet, inside a high vacuum chamber, with base pressure in the 10^–8^ Torr range. During the experiments, the chamber pressure was kept below 10^–5^ Torr, and the gas needle was grounded. The emergent beam was recorded by a light-sensitive diode.

The ionized recoil fragments produced by the interaction of the gaseous sample with the light beam are accelerated by a two-stage electric field and detected by a pair of microchannel plate detectors mounted. Electrons, accelerated in an opposite direction with respect to the positive ions, are recorded without energy analysis by two microchannel plate detectors and provide the start signal to the TDC. The ions produce stop signals to a time-to-digital converter (TDC). A DC electric field of 708 V/cm is applied to the first ion acceleration stage. The time-of-flight spectrometer was designed to have 100% efficiency for ions with kinetic energies up to 30 eV.^16^ The electrons produced in the ionization region are focused by an electrostatic lens biasing the electron grid with 800 V, designed to focus them at the center of the microchannel plate detector. Negative ions may also be produced and detected, but the corresponding cross sections are negligible. A commercially obtained racemic mixture was used in this work. HC_2_BrClF_3_ (Aldrich, 99% purity) was used after several freeze–pump–thaw cycles. We obtained conventional time-of-flight mass spectra from the correlation between one electron and an associated positive fragment (PEPICO).

## Computational Details

The ground-state geometry of the halothane molecule was obtained by applying *ab initio* calculations using the Møller–Plesset perturbation theory (MP2) and the aug-cc-pVTZ basis set via the quantum mechanical computational package Gaussian 2009. Our main results concerning excited-state properties were obtained with the Molpro quantum chemistry package,^20^ as explained in what follows. To obtain the transition energies and squared transition dipole moments, a series of computations with multiconfigurational self-consistent field for inner-shell states (IS-MCSCF)^21^ were performed followed by a multireference configuration interaction (MRCI) method. The cc-pVTZ basis set has been used in conjunction with scalar relativistic effects using Douglas–Kroll–Hess Hamiltonian (i.e., cc-pVTZ-DK) up to third order except for the atoms whose core orbitals are involved in the transitions for which the aug-cc-pVTZ-DK basis set was applied instead. The IS-MCSCF protocol was extensively applied for small halogenated organic molecules.^22−25^

**Figure 1:**
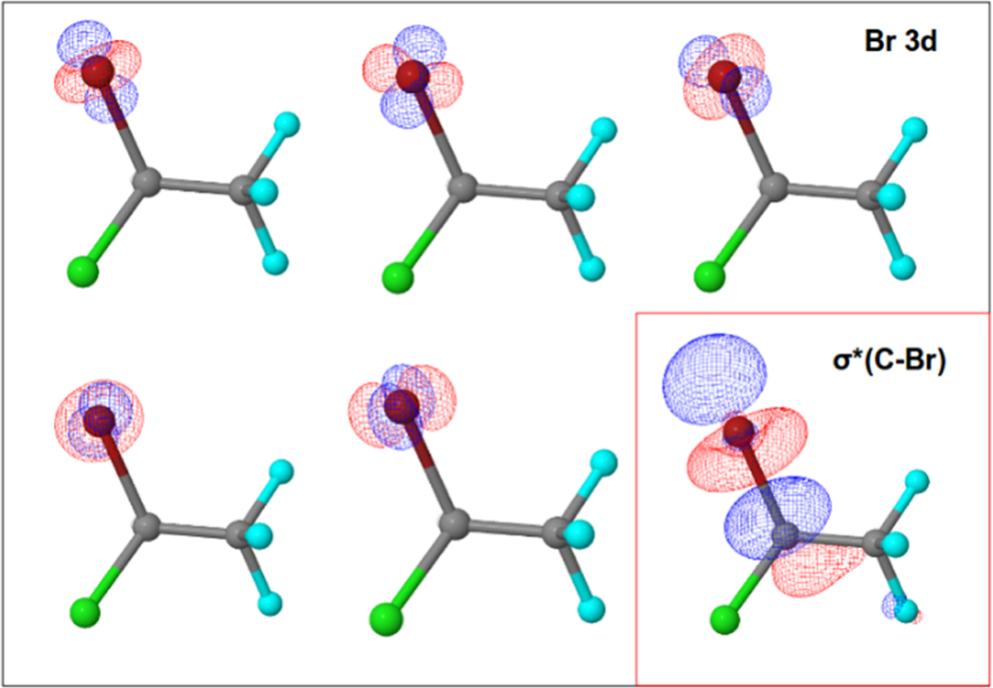
Active space composed of five Br 3d orbitals and σ* (C–Br) molecular orbital for Br 3d → σ* (C–Br) transition. Bromine atom is red, chlorine atom is green, fluorine atoms are blue, carbon atoms are gray, and hydrogen atom is white (not showing).

**Figure 2:**
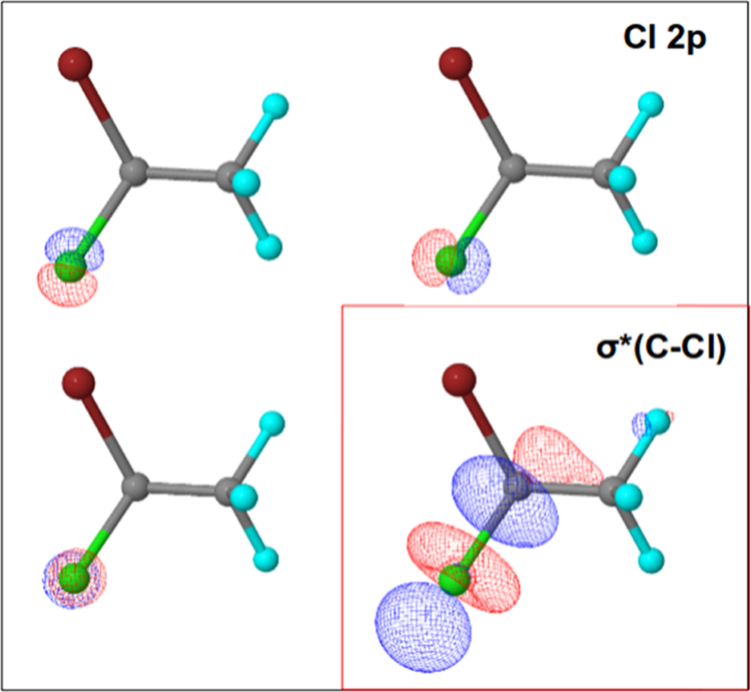
Active space composed by three Cl 2p orbitals and σ* (C···Cl) molecular orbital for Cl 2p → σ* (C–Cl) transition.

**Figure 3:**
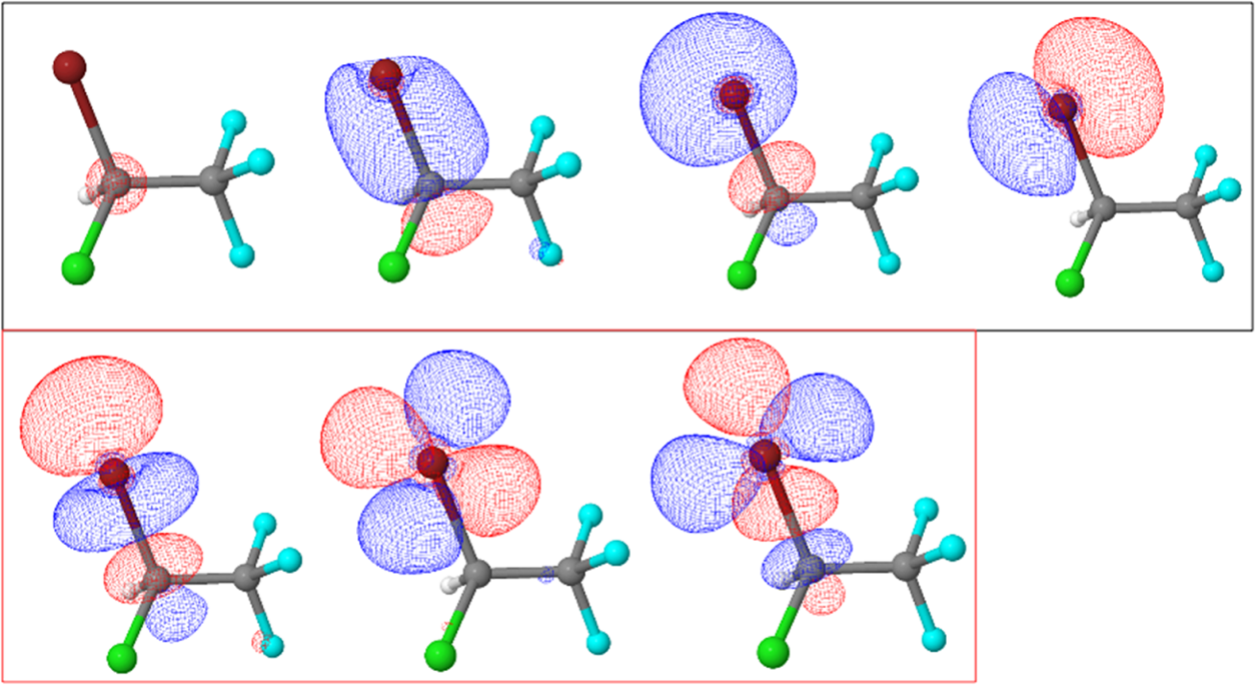
Active space composed of one C 1s, three occupied, and three virtual molecular orbitals for the C(1) 1s → σ* transition.

**Figure 4:**
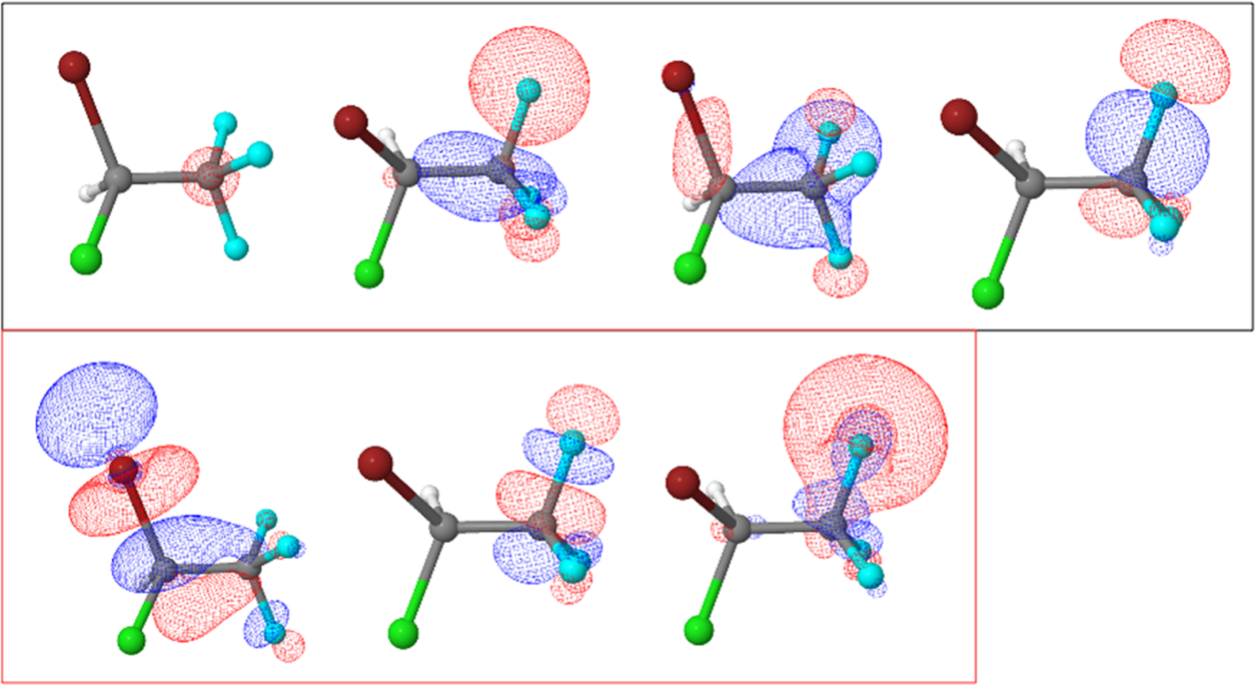
Active space composed of one C 1s, three occupied, and three virtual molecular orbitals for C(2) 1s → σ* transition.

In the MRCI step, state-averaged orbitals were used to construct a set of singlet and triplet states at the Cl 2p and Br 3d excitation edges, which will be used as the basis for the full Breit–Pauli Hamiltonian diagonalization.^26^ The C 1s edge was also considered, and the IS-CASSCF protocol was applied^27−29^ considering the different active spaces for each carbon atom. The orbitals chosen to compose the active space in the MCSCF calculations are shown in Figures 1–4. Active spaces were composed of five 3d orbitals of Br and σ* (C–Br) molecular orbitals for the Br 3d → σ* (C–Br) transition, three 2p orbitals of Cl and σ* (C–Cl) molecular orbitals for the Cl 2p → σ* (C–Cl) transition, and two 1s orbitals of C and σ* molecular orbital. This simple wave function can be considered as a state-averaged Hartree–Fock. This procedure has been previously successfully applied in the study of S 1s excitation in a different molecule.^30^ For the C 1s → σ* transitions, the 1s (C) core orbital, three occupied, and three virtual molecular orbitals were considered in the active spaces (Figures 3 and 4). Carbon one (C(1)) is the carbon atom bonded to Br and Cl atoms and carbon two (C(2)) is the carbon atom bonded to F atoms.

Besides the IS-MCSCF calculations, time-dependent density functional theory (TDDFT) was also applied for C 1s → σ* transition with PBE0 functional and same basis set using ORCA 4.2.1 quantum chemistry program package.^31^ All transitions were restricted from the two (localized) C 1s orbitals to the virtual ones.

## Results

### Total Ion Yield

**Figure 5:**
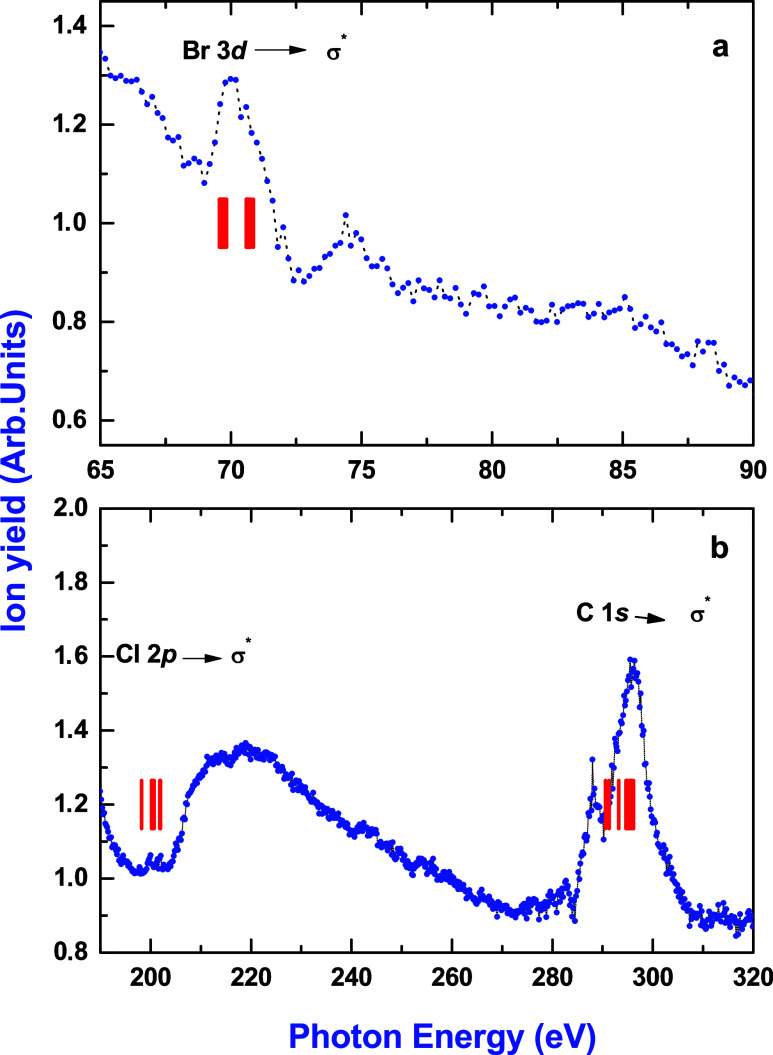
Wide-range energy scan of halothane total ion yield, i.e., the total number of ions produced, showing Cl 2p and C 1s edges (Figure 5b, bottom) and Br 3d (Figure 5a, top). The vertical red lines indicate the calculated transitions from Tables 1–3. Theoretical values for Cl 2p and C 1s have been shifted by +4 and +12 eV, respectively, to match the experimental values.

Absorption measurements are of paramount importance in the determination of the electronic or geometric structures of molecules. The assignment of the bands is done along with the discussion of the theoretical calculations. The wide-range total ion yield spectrum, which mimics the photoabsorption spectrum, was measured around the Br 3d, Cl 2p, and C 1s edges and is shown in Figure 5. Tables 1–3 present the unshifted transition energies and optical oscillator strengths for the Br 3d → σ* (C–Br), Cl 2p → σ* (C–Cl), and C 1s → σ* transitions, respectively.

**Table 1:** Unshifted Transition Energies and Squared Transition Dipole Moment of the Br 3d → σ* (C–Br) Transition^a^

transition	transition energy (eV)	transition dipole moment (*D*^2^)
Br 3d → σ* (C–Br)	64.56	0.00008
	64.56	0.00022
	64.57	0.00004
	64.60	0.00001
	64.61	0.00054
	64.63	0.00023
	64.63	0.00053
	64.64	0.00009
	64.69	0.00049
	64.72	0.02548
	64.76	0.02449
	64.83	0.00400
	65.59	0.00052
	65.60	0.00180
	65.63	0.00003
	65.64	0.00002
	65.68	0.00042
	65.71	0.02275
	65.75	0.02762
	65.86	0.00784

a States with same energies are degenerate.

**Table 2:** Unshifted Transition Energies and Squared Transition Dipole Moment of the Cl 2p → σ* (C–Cl) Transition^a^

transition	transition energy (eV)	transition dipole moment (*D*^2^)
Cl 2p → σ* (C–Cl)	196.19	0.00000
	196.19	0.00029
	196.20	0.00029
	196.28	0.00000
	196.28	0.00000
	196.38	0.00664
	196.44	0.00727
	196.70	0.00390
	197.85	0.00000
	197.92	0.00485
	197.96	0.00584
	198.24	0.00587

a States with same energies are degenerate.

**Table 3:** Unshifted Transition Energies and Optical Oscillator Strength of the C 1s → σ* Transition Obtained with the PBE0 Functional

transition	transition energy (eV)	optical oscillator strength (OOS)
C 1s → σ*	278.57	0.04696
	279.27	0.04598
	281.21	0.01318
	282.66	0.00390
	282.79	0.00462
	282.89	0.01006
	283.04	0.00267
	283.41	0.00041
	283.48	0.01905
	283.6	0.01010
	283.83	0.00110
	284.14	0.00254
	284.52	0.00092
	284.57	0.00576
	284.81	0.01813
	285.41	0.00799
	285.77	0.02167
	285.81	0.00367
	285.95	0.00929
	286.18	0.00401

In Figure 5, the two structures observed in the extended total ion yield spectrum around 300 eV (bottom) are displayed on top of a background due to the Cl 2p continuum. The characteristic features of the spin–orbit splitting resolved Cl 2p excitation can be observed in the figure as the 2p → σ* resonance (200.1 and 202.3), which, in turn, is embedded in the Br 3d and 3p continua. For the Br 3d edge, the structure at 70.1 eV is embedded in the continuum due to the direct photoionization of valence electrons. At the Br 3d → σ* resonance, we observe a shoulder due to not completely resolved spin–orbit splitting. The resonant Auger process, a nonradiative scattering event, involves an incoming photon exciting the target through dipole interaction. Subsequently, the decay results in the emission of an electron due to Coulomb interaction.^32^

The highest occupied molecular orbital (HOMO) and the second highest occupied molecular orbital (HOMO – 1) of halothane have Br 4p lone pair character,^14^ whereas the HOMO – 2 (44a) has Cl 3p lone pair character. The lowest unoccupied molecular orbitals, 47a (LUMO) and 48a (LUMO + 1), are mainly of σ*(C–Br) and σ*(C–Cl) antibonding character.

**Figure 6:**
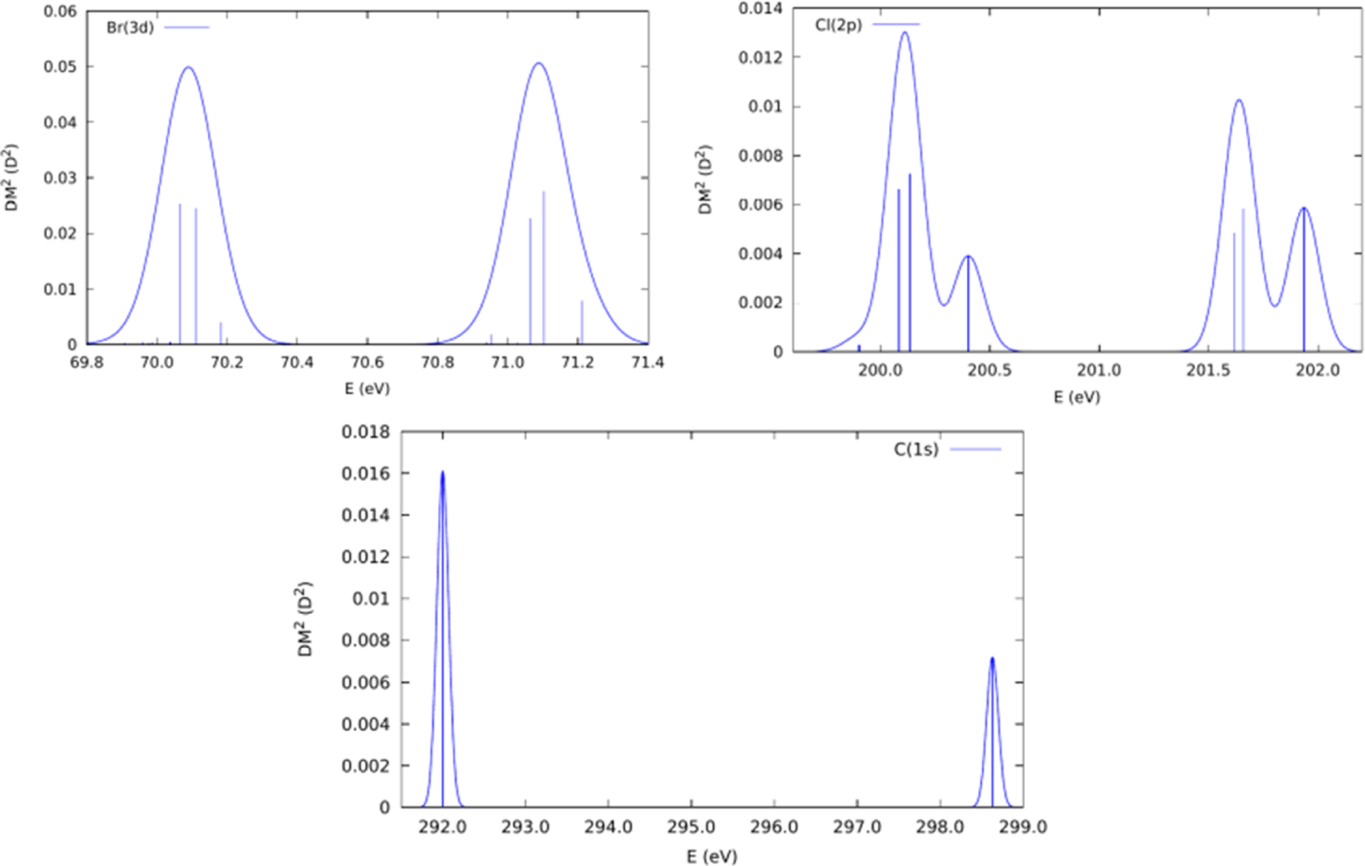
Simulated absorption spectra for Br 3d → σ* (C–Br) (top left), Cl 2p → σ* (C–Cl) (top right), and C 1s → σ* (bottom) transitions. Gaussian broadening curves with full width at half-maximum (fwhm) values of 0.1 eV and vertical shifts of 5.35, 3.7, and 3.25 eV for Br 3d → σ* (C–Br) (top left), Cl 2p → σ* (C–Cl) (top right), and C 1s → σ* (bottom) transitions were applied, respectively.

For the Br 3d → σ* (C–Br) transition, active orbitals are five 3d Br atomic orbitals and a sigma antibonding molecular orbital along the C–Br bond (σ* (C–Br)). A total of 20 states compose the basis for the full Breit–Pauli (BP) Hamiltonian diagonalization. Despite the large number of states, only four transitions show significant dipole moments (Table 1) with transition energies around 70.1 and 71.1 eV (Figure 6, top left panel) resulting in an energy splitting of the order of 1.0 eV. These results indicate that when the electron is excited to the σ* (C–Br) molecular orbital, the C–Br bond is weakened, and its cleavage resulting in the release of a Br atom from the molecular structure is facilitated. This trend was observed in previous works,^1,24^ that is, the electronic transition to the σ* orbital generates a dissociative state with the consequent selective cleavage of the carbon–halogen bond.

Furthermore, the active orbitals for the Cl 2p → σ* (C···C) transition are three 2p Cl atomic orbitals and a sigma antibonding molecular orbital along the C···Cl bond (σ* (C···Cl)). In this case, 12 states compose the basis for the BP Hamiltonian and 6 transitions have appreciable squared transition dipole moments. Around 200.1 and 201.6 eV (Figure 6, top right panel), two intense bands are present in the simulated absorption spectrum. Two small bands can also be identified at 200.4 and 201.9 eV revealing a more complex spectrum than the one observed in the Br 3d → σ* (C–Br) case. As observed at the Br 3d edge, when the electron is excited to the σ* (C–Cl) molecular orbital, the carbon–chlorine bond should be weakened, facilitating the bond cleavage. The corresponding transition energies are presented in Table 2.

Regarding the C 1s → σ* transitions, different active spaces were considered using the IS-CASSCF protocol in order to obtain the energy difference between the two possibilities, namely, C(1) 1s → σ* and C(2) 1s → σ*. The first transition is observed at 292 eV from the C 1s orbital of the carbon atom bonded to Br and Cl (C(1)) atoms. The transition energy from the C 1s orbital of C(2) (carbon atom bonded to F atoms) is 298.6 eV, resulting in an energy difference of 6.6 eV (Figure 6, bottom panel).

**Figure 7:**
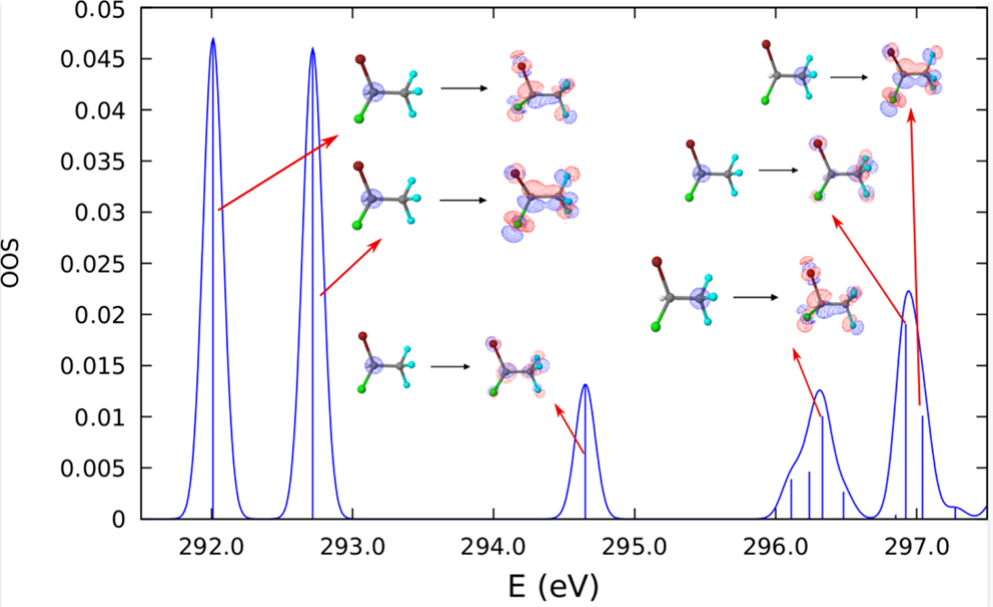
Optical oscillator strength (OOS) as a function of the photon energy. Simulated (TDDFT) absorption spectra for C 1s → σ* (bottom) transitions using the PBE0 functional. Gaussian broadening curves with full width at half-maximum (fwhm) values of 0.1 eV and vertical shifts of 13.4 eV were applied.

The results obtained with TDDFT agree with those obtained with IS-CASSCF. The first bands are related to transitions originating in the C 1s orbital of the C(1) atom. The first transition that originates from the C 1s orbital of C(2) is the eighth one around 296.4 eV. The second transition from the same orbital appears slightly above 297 eV. Simulated absorption spectra and transition energies are in available in the Supporting Information (Figure 7 and Table 3).

### Mass Spectra

**Figure 8:**
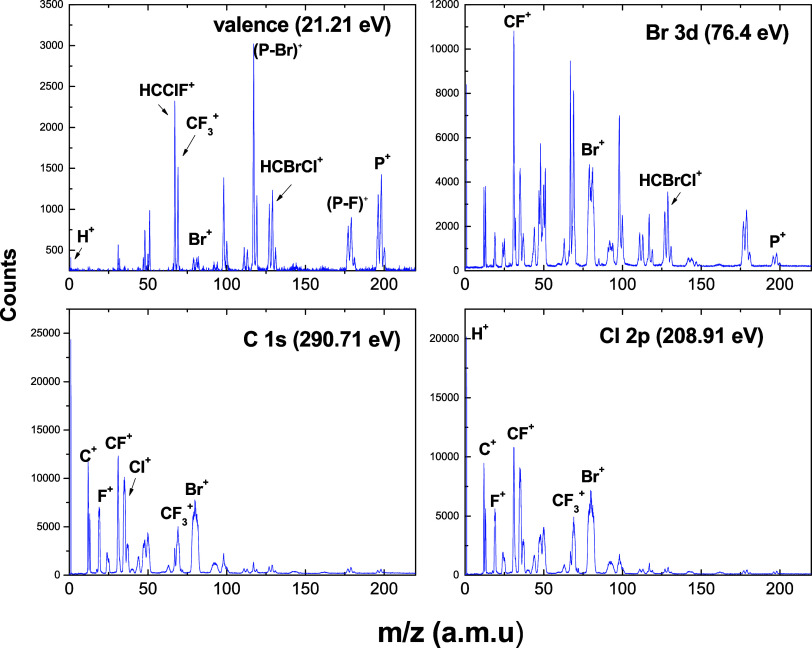
Time-of-flight mass spectra of the HC_2_BrClF_3_ molecule at selected photon energies.

The lowest experimental ionization energy of the halothane molecule occurs at *I*_p_ = 11.2 eV.^15,33^ If the double ionization threshold follows the empirical law 2.8*I*_p_,^34^ the expected double ionization apparent potential should be of the order of 31.36 eV. Figure 8 shows the mass spectra (PEPICO) of halothane at selected photon energies. In agreement with Marotta, Scorrano, and Paradisi,^35^ the major products observed at the 21.21 eV photoabsorption spectrum of halothane are the singly ionized parent (P^+^) molecule, with 18.5% relative abundance, the ion CF_3_CHCl^+^ (P–Br)^+^ (parent molecule reduced by the mass of Br), with 18.1% relative abundance (*m*/*z* = 117, 119) arising from C–Br bond cleavage, and the ion CHBrClCF_2_^+^ (12.4% relative abundance, *m*/*z* = 177, 179, 181) arising from the C–F Bond cleavage. Another intense signal observed in the time-of-flight spectrum is associated with the C–C bond cleavage, giving rise to the ion CHBrCl^+^ (12.5%, *m*/*z* = 127, 129,131). It is interesting to note that the moiety corresponding to the loss of a chlorine atom (*m*/*z* = 161) is absent from the mass spectrum at 21.21, and only a very weak signal (<0.6%) is observed at higher photon energies.

Considering the major products around the Br 3d edge (76.4 eV) of halothane are the parent molecular ion (18.5% relative abundance) and the ions CCl^+^, HCCl^+^, and CF_2_^+^ (12.9% relative abundance, *m*/*z* = 117, 119), the ion HCBrCl^+^ (12.5%, *m*/*z* = 127, 129,131) arising from a C–C Bond cleavage, and the ion HC_2_ClBrF_2_^+^ (12.4% relative abundance, *m*/*z* = 177, 179, 181) arising from C–F Bond cleavage. Additional intense signals in the mass spectrum correspond to the products of C–C bond rupture, HC_2_ClF_2_^+^ (8.6%, *m*/*z* = 99,100). The intensity of the ion CF_3_^+^ is considerable (5.3% at 21.21 eV) and does not change significantly with increasing photon energy.

Considering that the valence electrons are, contrarily to core electrons, delocalized over the molecule, the valence and core level photoionization mass spectra are expected to exhibit different patterns due to the distinct pathways leading to molecular fragmentation. As the photon energy increases to values related to the Cl 2p and C 1s resonances, we observe a broadening in the resonance peak widths. This effect can be understood in terms of the role played by the repulsive electronic states in the fragmentation of the core-excited molecules. The antibonding character of the final σ* orbital is responsible for the repulsive core-excited states. The exceeded energy is distributed as the kinetic energy shared by the fragments, as can be seen in Figure 8 for several fragments. For instance, in the case of Br^+^, which peak presents a significant broadening at C 1s (290. 71 eV) and Cl 2p (208.91 eV) edges.

At photon energies near the Br 3d edge and above, the mass spectra of halothane are dominated by a structure (∼21%) between masses (*m*/*z*) 78 and 81 corresponding to the C_2_^35^ClF^+^, C_2_^37^ClF^+^, ^79^Br^+^, ^81^Br^+^, HC_2_^35^ClF^+^, and HC_2_^37^ClF^+^ fragments. Above the Cl 2p edge, the structure between masses 91 and 94 corresponding to C^79^Br^+^, C^81^Br^+^, HC^79^Br^+^, and HC^81^Br^+^ is the second most prominent peak (∼15%) in the mass spectra. The structure between masses 47 and 50, corresponding to the CCl^+^, HCCl^+^, and CF_2_^+^, also plays an important role in the mass spectra of halothane, reaching its maximum around the Br 3d edge and decreasing slightly as a function of the photon energy.

**Figure 9:**
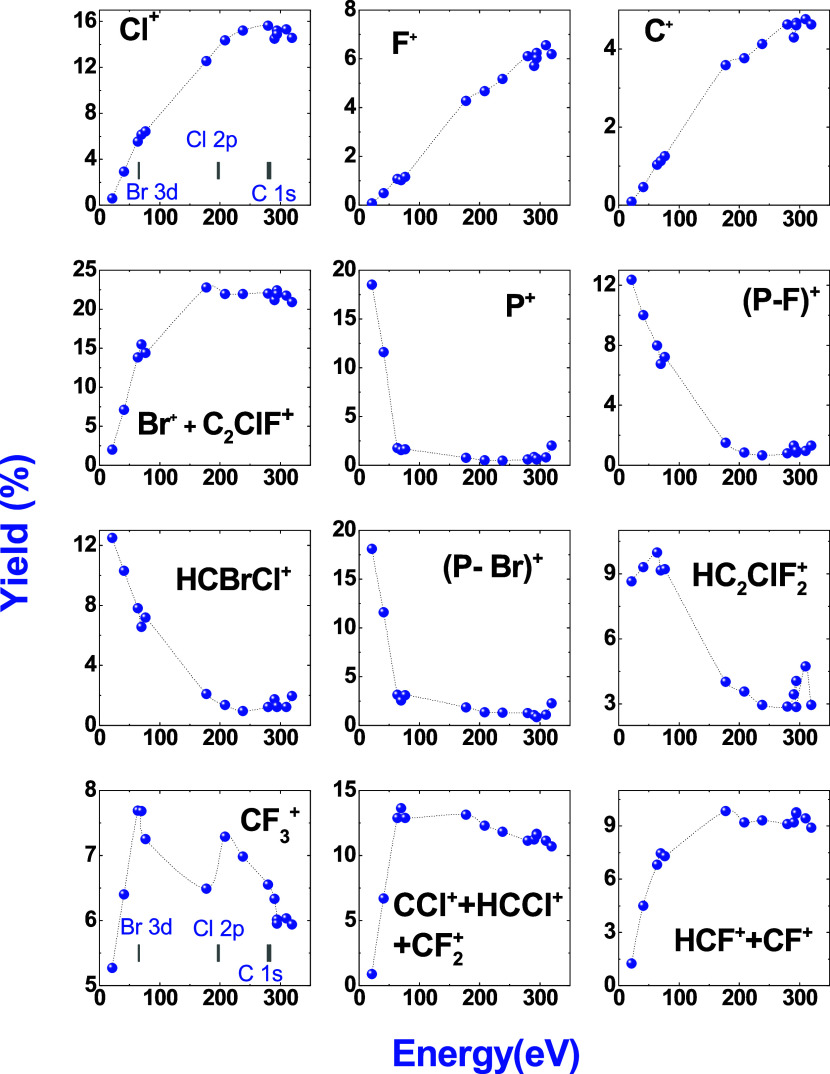
Ion yield percentage of some PEPICO fragments as a function of the photon energy.

The yield percentage of the fragments C^+^, F^+^, Cl^+^, C_2_ClF^+^, Br^+^ + HC_2_ClF^+^ + HBr^+^ + HC_2_F_3_^+^ + C_2_F_3_^+^ (*m*/*z* = 78–82) as well as the parent molecule (*m*/*z =* 196–200) and the fragment generated from the loss of one fluorine atom (*m*/*z* = 177–181) have been plotted as a function of the photon energy, and the results are shown in Figure 9.

Low-intensity (<1%) doubly charged fragments such as C_2_ClF^2+^ and Br^2+^ are observable above 70.2 eV. This indicates that the molecule fragments preferentially with charge separation instead of the asymmetric mechanism, in which one fragment retains the charge while the other remains neutral. Observation of the Br^2+^ (<1%) ion above the Br 3d edge may be due to the Auger decay process. We also observe an enhancement of the Cl^2+^ ion signal above the Cl 2p edge.

The integrated intensity of the CF_3_^+^ peak shows maxima at the Br excitation edge and above the Cl 2p edge, decreasing as the energy gets close to the C 1s excitation edge. This indicates a higher probability of C–C bond breakage in the cases of Br 3d excitation and Cl 2p ionization. Our hypothesis is that when a C 1s electron is excited or ionized, this favors the release of neutral F atoms provoking an increase in the fragmentation of the CF_3_^+^ ion.

The observed differences in the fragmentation pattern at the Br 3d, Cl 2p, and C 1s edges can be understood as follows. Following electronic relaxation through Auger decay produces unstable molecular ions that then dissociate. The outer-shell electrons that take part in the relaxation process come from molecular orbitals that overlap with the excited inner-shell orbital. Consequently, the primary Auger decay has a strong local aspect. Ions are then produced in a characteristic state, giving rise to a peculiar fragmentation pattern. More specifically, the selectivity could be ascribed to LVV (Cl 2p edge), MVV (Br 3d edge), or KVV (C 1s edge) Auger decays in which the hole is restricted to the corresponding excited element.

**Figure 10:**
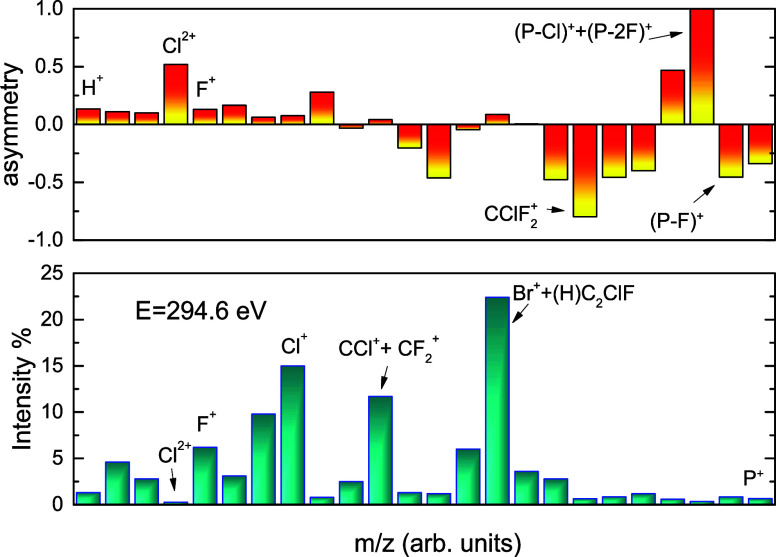
Top graph: asymmetry parameter (eq 1) for the site-selective excitation of halothane around the C 1s edge. Bottom graph: Relative intensities of the halothane fragments were 294.6 eV.

In order to search for a possible site selectivity due only to core excitation of the C(1) and C(2) carbon atoms, we subtracted from the mass spectra the contribution from the background continua (valence + Br 3d + Cl 2p). In this procedure, we normalize the mass spectra obtained at 290.7 and 294.6 eV, corresponding to C(1) and C(2) ionization, respectively, to the corresponding ion yield and subtract the background continua (valence + Br 3d + Cl 2p) from the normalized mass spectra taken at 280 eV (below the C 1s edge). In the next step, we introduce the asymmetry parameter for the evaluation of the site specificity in fragmentation.
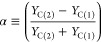
1where Y_C(1)_ and Y_C(2)_ are the normalized yields for each fragment after background subtraction. Note that a 100% site specificity for a fragment results in α = ±1. Figure 10 shows the asymmetry parameter for the two carbon sites of halothane and their respective yields at 294.6 eV (carbon C(2)). We clearly note from the data that most fragments show weak site specificity. The fragments with larger α values are those with low intensities. The mean value for the absolute asymmetry parameter is ⟨|α|⟩ = 0.29 ± 0.26, and the weighted mean (weighted with respect to the relative intensities of the fragments) is ⟨|α|⟩_w_ = (4.9 ± 4.3) × 10^–3^. This means that the halothane molecule exhibits very low site specificity in its ionic dissociation, following core ionization. In other words, this molecule virtually loses the “memory” of the photoexcited site.

In general, site specificity is understood as being induced either by Auger relaxation, whose outcome depends on the initial excitation site, for instance, (C(1) or C(2)), or by a dependence on the fragmentation pattern from specific energy states of the singly charged molecule on the initial charge localization. Fragmentation of the halothane molecule following C 1s excitation is quite nonspecific with respect to the initially localized carbon atom (C(1) or C(2)). Usually, this is explained in terms of an equalization of internal energy into vibrational degrees of freedom after Auger decay.^4,9,36,37^

## Summary and Conclusions

In this article, we present and discuss a PEPICO study in a broad excitation energy range extending from the bromine 3d valence, chlorine 2p, and carbon 1s edges of the halothane molecule. In addition, *ab initio* quantum mechanical calculation at the MP2 level and aug-cc-pVTZ basis set and a series of computations with multiconfigurational self-consistent field for inner-shell states (IS-MCSCF) were used to calculate the oscillator strengths for the transitions. Outer and inner-shell photoionization give rise to a variety of fragmentation patterns that depend on the photon energy and the nature of the selected core level. The mass spectra obtained in the present work, at the Br 3d, Cl 2p, and C 1s edges, revealed significantly different ion yield profiles.

The presence of two carbon atoms in distinct chemical environments is an opportunity to study site selectivity. Notwithstanding, very weak site selectivity was observed on the ionic photofragmentation of the halothane molecule excited at the C 1s edge. Only high-mass molecular fragments (*m*/*z* > 80) exhibit a significative asymmetry due to site selectivity. However, the relative low intensities of those fragments do not significantly contribute to the overall site selectivity of the molecule. This is due, probably, to an equalization of internal energy into vibrational degrees of freedom after Auger decay.

## Supporting Information Available

Table containing the branching ratios for the detected fragments (PDF)
