# Supplementary material for: Ionic Fragmentation of the Halothane Molecule Induced by EUV and Soft X-ray Radiation
Source: J Phys Chem A. 2024 Aug 23;128(35):7407–16. doi: 10.1021/acs.jpca.4c04341 (PMC11382275; doi:10.1021/acs.jpca.4c04341)
Supplement: Supplementary file 1 — jp4c04341_si_001.pdf [file jp4c04341_si_001.pdf]

Ionic Fragmentation of the Halothane Molecule induced by EUV and soft X-ray radiation

A.C. F. Santos[1*], C. A. Lucas[2 **], A. F. Lago[3***], R. R. Oliveira[4#], A. B. Rocha[4##], G. G. B. de Souza[4###],

[1]Instituto de Física, Universidade Federal do Rio de Janeiro (UFRJ), Ilha do Fundão, Rio de Janeiro, RJ, Brazil, 21949-900

[2]Instituto de Química, Universidade Federal Fluminense, Outeiro de São João Batista s/n, Campus do Valonguinho, Niterói, Brazil, 24020-141

[3]Centro de Ciências Naturais e Humanas, Universidade Federal do ABC (UFABC), Av. dos Estados, 5001, 09210-580, Santo André, SP, Brazil.

[4]Instituto de Química, Universidade Federal do Rio de Janeiro (UFRJ), Ilha do Fundão, Rio de Janeiro, RJ, Brazil, 21949-900

*Corresponding author: toni@if.ufrj.br

** carloslucas@id.uff.br
***alexsandre.lago@ufabc.edu.br
rrjunior@pos.iq.ufrj.br
rocha@iq.ufrj.br
ggersonbs@yahoo.com

Supporting Information

M/q	Atribution	Intensity (%)												
		Synchrotron Radiation Energy (eV)												
		**21.21**	**40.8**	**64**	**70.22**	**76.4**	**178**	**208.9**	**238**	**280**	**290.7**	**294.6**	**310**	**320**
**1**	**H^+**	0.06	0.18	0.33	0.37	0.40	1.1	1.1	1.2	1.3	1.2	1.3	1.3	1.3
**12**	**C^+, C_2^{2+}**	0.08	0.46	1.0	1.1	1.3	3.6	3.8	4.1	4.6	4.3	4.6	4.8	4.6
**13**	**HC^+**	0.09	0.66	1.2	1.4	1.4	2.7	2.5	2.7	2.7	2.6	2.8	2.7	2.6
**17,5 and 18,5**	**$^{35,37}Cl^{2+}$**	-	-	0.09	-	-	0.06	0.15	0.22	0.34	0.25	0.27	0.33	0.31
**19**	**F^+**	0.06	0.48	1.1	1.0	1.2	4.3	4.7	5.2	6.1	5.7	6.2	6.6	6.2
**24<br>25**	**C_2^+, $HCCl^{2+}$(35) HC_2^+, CF_2^{2+}, $HCCl^{2+}$(37)**	-	-	1.3	1.6	1.6	2.9	2.8	2.8	2.8	2.7	3.1	2.9	2.8
**31<br>32**	**CF^+<br>HCF^+**	1.3	4.5	6.8	7.4	7.3	9.8	9.2	9.3	9.1	9.2	9.8	9.4	8.9
**35,37**	**$^{35,37}Cl^+$**	0.56	2.9	5.5	6.1	6.4	12.5	14.3	15.2	15.6	14.5	15.0	15.3	14.6
**40**	**$C_2Cl(37)F^{2+}$, Br^{2+}**	-	-	-	0.05	0.02	0.43	0.62	0.71	0.88	0.72	0.79	0.91	0.82
**44**	**HC_2F^+**	0.15	1.6	2.2	2.4	2.4	2.8	2.6	2.5	2.3	2.5	2.5	2.3	2.3
**47,48, 49, 50**	**CCl^+, $HCCl^+$, CF_2^+,**	0.89	6.7	12.9	13.6	12.9	13.1	12.3	11.8	11.1	11.2	11.7	11.1	10.7
**63**	**$HC_2F_2^+$**	0.21	0.97	1.8	1.7	1.7	1.4	1.4	1.4	1.2	1.5	1.3	1.2	1.2
**67**	**$HCCl(35)F^+$**	7.4	6.4	5.3	5.4	4.9	2.1	1.6	1.4	1.3	1.8	1.2	1.9	1.5
**69**	**CF_3^+**	5.3	6.4	7.7	7.7	7.3	6.5	7.3	7.0	6.6	6.3	6.0	6.0	5.9
**78, 79 80, 81 82**	**C_2ClF^+, Br^+, HC_2ClF^+, $HC_2F_3^+$**	2.00	7.1	13.8	15.5	14.4	22.8	21.9	21.9	22.0	21.1	22.4	21.7	20.9
**91,92 93,94**	**CBr^+, $HCBr^+$,**	0.74	2.3	3.8	3.5	3.9	2.5	3.6	3.6	3.3	3.5	3.6	3.3	3.2
**98, 100**	**$HC_2ClF_2^+$**	8.6	9.3	10.0	9.1	9.2	3.6	2.9	2.9	3.4	4.1	2.8	4.7	2.9
**111, 113**	**$CClF_2^+$**	3.9	3.7	3.5	3.1	3.3	0.95	1.2	0.92	0.91	1.4	0.64	0.73	1.0
**117, 119**	**$HC_2F_3Cl^+$**	18.1	11.6	3.1	2.6	3.1	1.8	1.3	1.3	1.3	1.1	0.85	1.1	2.2
**127, 129, 131**	**$HCBrCl^+$**	12.5	10.3	7.8	6.6	7.2	2.1	1.4	0.94	1.2	1.7	1.2	1.2	2.0
**142, 144**	**$HC_2Br(79)F_2^+$,**	2.7	2.1	1.3	1.2	1.4	0.31	0.79	0.67	0.57	0.46	0.59	0.58	0.53

	**$HC_2Br(81)F_2^+$**													
**161, 162, 164**	**$HC_2Cl(37)Br($ 81)F^+, $CBrF_3^+$, $HC_2Br(79)F_3^+$, $CBr(79)ClF_2^+$**	-	-	0.20	0.20	0.19	-	0.57	0.48	0.42	0.23	0.34	0.34	0.34
**177, 179, 181**	**$HC_2ClBrF_2^+$ P – F**	12.4	10.0	8.0	6.8	7.2	1.5	0.84	0.64	0.79	1.3	0.84	0.94	1.3
**196, 198, 200**	**$HC_2ClBrF_3^+$ P**	18.5	11.6	1.7	1.5	1.6	0.76	0.52	0.45	0.57	0.87	0.65	0.78	2.0

Table S1 – Relative Intensities of the fragments of halothane as a function of the photons energy.
